# *Pax2/5/8* and *Pax6* alternative splicing events in basal chordates and vertebrates: a focus on paired box domain

**DOI:** 10.3389/fgene.2015.00228

**Published:** 2015-07-02

**Authors:** Peter Fabian, Iryna Kozmikova, Zbynek Kozmik, Chrysoula N. Pantzartzi

**Affiliations:** Department of Transcriptional Regulation, Institute of Molecular GeneticsPrague, Czech Republic

**Keywords:** Pax258, Pax6, alternative splicing, paired domain, splice variants

## Abstract

Paired box transcription factors play important role in development and tissue morphogenesis. The number of *Pax* homologs varies among species studied so far, due to genome and gene duplications that have affected PAX family to a great extent. Based on sequence similarity and functional domains, four Pax classes have been identified in chordates, namely Pax1/9, Pax2/5/8, Pax3/7, and Pax4/6. Numerous splicing events have been reported mainly for *Pax2/5/8* and *Pax6* genes. Of significant interest are those events that lead to Pax proteins with presumed novel properties, such as altered DNA-binding or transcriptional activity. In the current study, a thorough analysis of *Pax2/5/8* splicing events from cephalochordates and vertebrates was performed. We focused more on *Pax2/5/8* and *Pax6* splicing events in which the paired domain is involved. Three new splicing events were identified in *Oryzias latipes*, one of which seems to be conserved in Acanthomorphata. Using representatives from deuterostome and protostome phyla, a comparative analysis of the *Pax6* exon-intron structure of the paired domain was performed, during an attempt to estimate the time of appearance of the *Pax6(5a)* mRNA isoform. As shown in our analysis, this splicing event is characteristic of Gnathostomata and is absent in the other chordate subphyla. Moreover, expression pattern of alternative spliced variants was compared between cephalochordates and fish species. In summary, our data indicate expansion of alternative mRNA variants in paired box region of *Pax2/5/8* and *Pax6* genes during the course of vertebrate evolution.

## Introduction

Transcription factors encoded by genes of the paired box (PAX) family are highly conserved throughout metazoan phyla and hold a vital role in embryonic development. The association of different PAX subfamilies with organ and tissue morphogenesis, such as the thymus, central nervous system (CNS), enteric nervous system, kidneys, ear, thyroid, neural crest, vertebrae, and midbrain-hindbrain boundary (MHB) formation has been the object of various studies (summarized in Noll, 1993; Chi and Epstein, 2002; Paixao-Cortes et al., 2013; Blake and Ziman, 2014). Certain members of the PAX family are characterized as the master control genes for eye morphogenesis (Gehring, 1996, 2002, 2012; Kozmik, 2008; Klimova and Kozmik, 2014) and genetic defects are linked to the onset of eye-related diseases, e.g., small eye in mouse or aniridia in human. Mutations in *Pax* genes are also correlated with diseases of the kidney and the CNS, but various types of cancer as well (see Chi and Epstein, 2002; Lang et al., 2007; Paixao-Cortes et al., 2013; Blake and Ziman, 2014 and references therein).

Gene and genome duplications, followed by gene losses, helped shape PAX gene family, leading to a varying number of *Pax* homologs in metazoan phyla studied so far (reviewed in Noll, 1993; Breitling and Gerber, 2000; Hoshiyama et al., 2007; Paixao-Cortes et al., 2013). Different subfamilies have been identified and are classified according to the similarity in sequence, functional domains they possess as well as their expression patterns (see Stuart et al., 1994; Blake and Ziman, 2014 and references therein). *PaxB* is apparently the oldest member and is present in sponges and cnidarians (Kozmik et al., 2003; Hoshiyama et al., 2007; Hill et al., 2010). *Pox neuro* and single genes from Pax1/9, Pax2/5/8, Pax4/6, and Pax3/7 classes are present in cephalochordates (Short and Holland, 2008; Takatori et al., 2008). *Pox neuro* is present in *Drosophila* (Bopp et al., 1989), but is lost in the lineages of tunicates and vertebrates. Two *Pax258* genes are present in urochordates, due to a duplication prior to ascidian and larvacean diversification (Wada et al., 2003; Canestro et al., 2005). As a result of two rounds of whole-genome duplication (Escriva et al., 2002; Putnam et al., 2008) and subsequent gene losses, the coelacanth *Latimeria chalumnae* possesses nine *Pax* genes, i.e., discrete gene copies from each of the Pax1/9, Pax2/5/8, Pax3/7, and Pax4/6 classes, suggesting that all members of the PAX family were present in the ancestor that gave rise to the tetrapod lineage (Paixao-Cortes et al., 2013). More than nine genes are present in teleost fishes (reviewed in Ravi et al., 2013), due to the so-called third round of genome duplication (Jaillon et al., 2004; Van De Peer, 2004).

All Pax proteins contain a DNA-binding domain in their N-terminus, known as the paired domain (PD), as well as transactivation and inhibitory domains in their C-terminus. The PD consists of 128 aminoacids and is made up by two helix-turn-helix (HTH) subdomains, known as PAI and RED or N-terminal and C-terminal respectively, joined through a linker (Czerny et al., 1993; Xu et al., 1999). The Pax4/6 class contains an additional DNA-binding homeodomain, while the Pax2/5/8 possesses a partial homeodomain and an octapeptide motif, the latter known to interact with members of the Groucho family of co-repressors (Eberhard et al., 2000; Kreslova et al., 2002). Classes Pax1/9 and Pax3/7 both contain the octapeptide motif, yet the former lacks the homeodomain (Chi and Epstein, 2002). *Pax* loci which encode for proteins with truncated paired domain have been identified in *Drosophila*, *C. elegans*, as well as representatives of Hemichordata and Echinodermata (Chisholm and Horvitz, 1995; Cinar and Chisholm, 2004; Howard-Ashby et al., 2006; Friedrich and Caravas, 2011; Ravi et al., 2013).

Gene/genome duplication is a driving force for evolution (Bergthorsson et al., 2007; Maere and Van De Peer, 2010) and this could be nicely exemplified by the well-studied PAX gene family, where many duplicates were preserved in the genome and obtained new functions and new domains of expression (neofunctionalization), or original gene functions were partitioned (subfunctionalization) between duplicates (Pfeffer et al., 1998; Bassham et al., 2008; Kleinjan et al., 2008, reviewed in Holland and Short, 2010).

At posttranscriptional level, alternative splicing is also known to promote evolution, protein diversity and development of novel functions in eukaryotic genomes (reviewed in Nilsen and Graveley, 2010; Chen et al., 2012; Kelemen et al., 2013). In fact, it has been suggested that in the case of *Pax* genes the impact of alternative splicing on functional motifs is more intense than gene duplication and subsequent divergence of the duplicated genes (Short and Holland, 2008). It has been shown that alternative spicing usually takes place in a tissue or developmental stage-specific manner (Wang et al., 2008a; Kelemen et al., 2013). Depending on which exonic segments are cut-out and whether intronic regions are retained in the transcripts, splicing events can be clustered into four major groups, namely (1) exon skipping, (2) alternative 3′-, (3) alternative 5′-splice sites, and (4) intron-inclusion (Koralewski and Krutovsky, 2011). These four types of events can occur independently or in combination with other incidents, such as mutually exclusive exons, alternative initiation and alternative polyadenylation (Wang et al., 2008a; Koralewski and Krutovsky, 2011; Kelemen et al., 2013).

Alternative splicing of *Pax* genes has been observed in various species, from protostomes (Fu and Noll, 1997; Cinar and Chisholm, 2004) to cephalochordates (Glardon et al., 1998; Kozmik et al., 1999; Short and Holland, 2008; Holland and Short, 2010; Short et al., 2012) and vertebrates (Kozmik et al., 1993, 1997; Poleev et al., 1995; Heller and Brandli, 1997, 1999; Lun and Brand, 1998; Short et al., 2012), where multiple incidents from all major groups of splicing events were present. In principle, splice forms seem to have diverged between lineages, some of them are species- or genus-specific (Heller and Brandli, 1999; Short et al., 2012), nevertheless several splice isoforms seem to be evolutionary conserved (Kwak et al., 2006; Short and Holland, 2008; Short et al., 2012; Ravi et al., 2013). The majority of reported splice events regards the transactivation and inhibitory domain in the C-terminal part of the Pax proteins (Kozmik et al., 1993; Ward et al., 1994; Nornes et al., 1996; Tavassoli et al., 1997; Kreslova et al., 2002; Robichaud et al., 2004), still there is an increasing number of events affecting paired domain and consequently DNA binding capacity (Kozmik et al., 1993, 1997; Zwollo et al., 1997; Short and Holland, 2008; Short et al., 2012). One such example is the *Pax6(5a)* isoform, where inclusion of exon 5a (Walther and Gruss, 1991; Glaser et al., 1992; Puschel et al., 1992) leads to a protein with interrupted paired domain that recognizes an altered DNA binding sequence (Epstein et al., 1994). Apparently, this event is quite conserved among vertebrate lineages, with differences in size and peptide sequence of exon 5a between fish and tetrapods (Ravi et al., 2013). In some cases, alternatively spliced *Pax* isoforms exhibit temporally and spatially differentiated expression patterns (Kozmik et al., 1993, 1997; Heller and Brandli, 1997; Short and Holland, 2008) and have been associated with cancer and genetic disorders (reviewed in Wang et al., 2008b; Holland and Short, 2010).

Previous studies have shown that any insertion in the conserved paired domain, no matter if it is a single-aminoacid extension or a whole exon cassette, modifies DNA binding capacity and attributes differentiated functions to the isoforms bearing the insertion (Kozmik et al., 1997; Azuma et al., 2005).

In the present study, we sought to identify splicing events in Pax2/5/8 and Pax6 classes that affect the paired domain and study the expression patterns of these alternative spliced transcripts. We identified three new splicing events in *Oryzias latipes Pax2* genes, one of which seems to be highly conserved in Acanthomorphata. We detected a re-occurring splicing event in *O. latipes and Danio rerio Pax6* genes generating the exon 5a insertion. Using our data set we tried to elucidate the time point at which the exon 5a-insertion appeared and the extent of its conservation in various phylogenetic groups.

## Materials and methods

### Data collection and *de novo* gene annotation

Nucleotide and aminoacid sequences for annotated *Pax2*/5/8 and *Pax6* genes were obtained using proper keywords, through NCBI GenBank (Benson et al., 2013), ENSEMBL release 78 (Cunningham et al., 2015), the UCSC Genome Browser database (Karolchik et al., 2014), the SpBase (Sea Urchin Genome Database, Cameron et al., 2009) and the JGI (Grigoriev et al., 2012). The retrieved *Pax* genes were crosschecked using GENSCAN (Burge and Karlin, 1997), BLASTx and version 0.9 of the NNSPLICE splice predictor (Reese et al., 1997).

For various taxonomic groups (e.g., Chondricthyes:Holopocephali) there are available genomes, but no *Pax* genes are annotated in public databases. In order to include representatives from these groups, we conducted BLAST searches against the NCBI GenBank and wgs subdivision (Trace archive), using known homologs from Deuterostomia species. Where required, small contigs or scaffolds were fused using Merger of the EMBOSS software suite (Rice et al., 2000) and gene structure was defined using GENSCAN (Burge and Karlin, 1997), BLASTx and splice predictor (Reese et al., 1997). ScanProsite (De Castro et al., 2006) was used to detect conserved functional domains in newly identified genes. In addition, adjacent genes of the *de novo* predicted *Pax* genes were also predicted/annotated and gene order was compared to known *Pax* syntenic regions through the Genomicus website v78.01 (Louis et al., 2013). In all cases, the NNSPLICE was used for the prediction of possible alternative acceptor and donator sites.

PipMaker (Schwartz et al., 2000) was used along with BLAST, in order to locate putative sequence conservation among species. Alignment of Pax6 paired domains from various species was performed using the MUSCLE algorithm (Edgar, 2004) included in Mega version 5.0 (Tamura et al., 2011).

### Expressed sequenced tags (ESTs) retrieval and analysis

In order to validate already annotated or newly predicted *Pax* homologs, BLAST searches were performed against the ESTs subdivision. Collected ESTs were aligned with predicted coding sequences from genome analyses and mRNA sequences—if available—in order to detect putative splicing events not recognized so far.

### Animal collection

Specimens of *Branchiostoma floridae* were collected from Old Tampa Bay, Florida, USA. Gametes were obtained and embryos were raised, as previously described (Holland and Yu, 2004). *B. lanceolatum* adults were collected in Banyuls-sur-Mer, France, prior to summer breeding season and raised in the lab until spawning. The spawning of males and females was induced by temperature shift (Fuentes et al., 2007). *B. lanceolatum* and *B. floridae* embryos were developed at 16°C and 26°C, respectively. Embryos of inbred strains of *Oryzias latipes* (Cab) and *Danio rerio* (AB) were used for all experiments. *O. latipes* and *D. rerio* embryonic stages were determined according to Iwamatsu (2004) and Kimmel et al. (1995). Housing of animals and *in vivo* experiments were performed after approval by the Animal Care Committee of the Institute of Molecular Genetics (study ID#36/2007) and in compliance with national and institutional guidelines (ID#12135/2010-17210).

### RNA isolation and reverse transcription

Total RNA was isolated from embryos using the Trizol reagent (Ambion). Random-primed cDNA was prepared in a 20 μl reaction from 500 ng of total RNA using SuperScript VILO cDNA Synthesis kit (Invitrogen).

### Screen for alternative splicing and RT-PCR analysis

cDNA was subjected to PCR using DreamTaq polymerase (Thermo Scientific) for 30 cycles under the following conditions: 1 min at 98°C, 30 s at 60°C, 30 s at 72°C. Primers for this analysis are provided in Table 1. PCR products were analyzed on 2.5% agarose gel and bands of interest were eluted, cloned to pCR-Blunt II (Invitrogen) and sequenced (GATC Biotech sequencing service, Germany).

**Table 1:** **Summary of primers used in RT-PCR reactions in the present study**.

**Species**	**Gene**	**Sequence 5′−>3′**
*Branchiostoma*	Pax258	F: AATGGGTGCGTGTCCAAGATT
		R: AACGCTGGGGATGTTCTCATT
	Pax46	F: GTCCCACGGCTGTGTCAGTAA
		R: TCGTTGTCACAAATGCCTTCC
*Oryzias latipes*	Pax2.1	F: GCAGCGGATCGTGGAGCTT
		R: GAACATAGTGGGGTTTTGGCGC
		R^*^: CTCTGAAATGCCTTCTGATAG
	Pax2.2	F: GCAGCGGATTGTGGAGCTG
		R: AAACATTGTTGGGTTTTGTCTT
	Pax6.1	F: CCACCAGGCAGAAAATAGTGGAACTT
		R: ATCTTGCTCACGCAGCCGTTGGAT
	Pax6.3	F: AACCAGCTCGGGGGGGTATTTGTG
		R: CTCCAATGGCCCGGGGACGG
*Danio rerio*	Pax2.1	F: ACCAGCTAGGAGGGGTGTTT
		R: CCAGGCGAACATTGTAGGAT
	Pax2.2	F: CGACAGCTGAGGGTCAGTC
		R: CGAACATGGTGGGATTTTGT
	Pax6.1a	F: CCCGACTCCACGAGACAGAAAATAGTT
		R: ACCCAAGATTTTACTCACGCAGCCGTTG
	Pax6.1b	F: CCGGACTCCACCAGACAGAAGATCGTC
		R: CCAGAATCTTGCTCACGCACCCATTC

For Branchiostoma species the same set of primers was used for each gene class amplified. Oryzias latipes Pax2.1 reverse primer marked with asterisk was used to amplify alternative exon 2a along with Pax2.1 forward primer.

## Results

### *Pax2/5/8* splicing events in chordates

**Figure 1:**
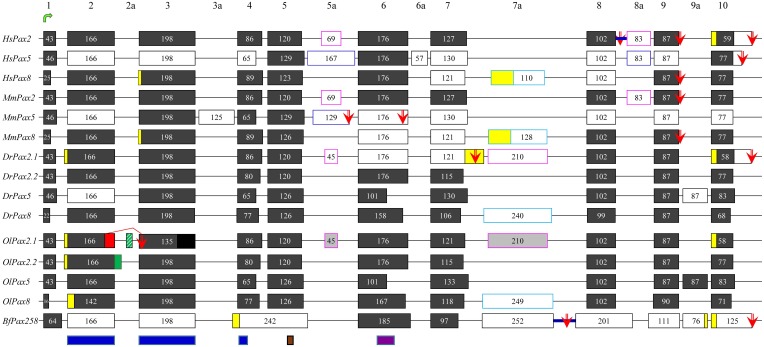
**Exon-intron organization and alternative splicing events of *Pax2/5/8* representatives from vertebrates and cephalochordates**. Dark gray and white boxes represent constitutive and alternatively spliced exons, respectively. Yellow boxes denote alternatively spliced parts of exons, due to different 5′ or 3′ splicing donors/acceptors. The suffix “a” is used for non-canonical exons, characteristic of the different *Pax2/5/8* genes (different colors of outline is used for different orthologs). For *O. latipes*, light gray boxes were predicted due to high similarity to respective exons in *D. rerio*, whereas black box shows the non-sequenced part of exon 3. Lines represent introns (not drawn to scale). Blue thick lines represent intron retention events. Green arrow points to exon containing translation initiation, while red arrows stand for alternative stop codons. Blue, brown, and purple boxes define borders of paired domain, the octapeptide, and partial homeodomain, respectively. Alternative splicing events detected in the present study are indicated by red and green boxes (alternative 5′ splice donors) and green stripped box (exon cassette). Previously published data are included (Dressler et al., 1990; Krauss et al., 1991; Kozmik et al., 1993, 1997, 1999; Ward et al., 1994; Poleev et al., 1995; Zwollo et al., 1997; Lun and Brand, 1998; Pfeffer et al., 1998; Borson et al., 2002; Robichaud et al., 2004; Kwak et al., 2006; Short and Holland, 2008; Arseneau et al., 2009; Busse et al., 2009).

Exhaustive search through databases and literature, in combination with *de novo* analysis of available ESTs and mRNA sequences (Table S1), revealed numerous splicing events in chordate members of the *Pax2/5/8* class (Figure 1). Some of these events seem to characterize specific orthologs and are present in cephalochordates, fish and mammals (e.g., exon 2 of *Pax5* gene), while others are much less conserved (e.g., exon 3a of mouse *Pax5*). *Branchiostoma floridae* appears to experience the largest number of splicing events, however no event of insertion in the paired domain has been reported so far and no such event could be predicted using splice prediction software or available ESTs/mRNA sequences.

A single *Oryzias latipes Pax2* gene, namely *OlPax2.2*, located on chromosome 19 (NC_019877), has been used in previous studies (Paixao-Cortes et al., 2013). Our search through NCBI revealed an annotated Pax-2a-like gene on chromosome 15 (NW_004088010.1). It must be noted that the aminoacid sequence encoded by the first half of this gene exhibits no similarity to the paired domain of other *Pax* genes and it is not supported by ESTs, a fact that indicates an erroneous gene prediction, caused by a non-sequenced area in this genomic region. We assume that the first coding exon as well as the two exons coding for the paired domain of *OlPax2.1* are located in this non-sequenced region. The record of an unplaced scaffold (NW_004093539, Table S1) was retrieved through BLAST. It apparently corresponds partly to the non-sequenced region of chromosome 15 and contains the 5′ UTR, the first exon of *OlPax2.1* gene and part of the first intron.

In order to retrieve more information on *OlPax2.1* and to elucidate how many *OlPax2* (either *Pax2.1* or *Pax2.2*) transcripts exist, we searched for different ESTs and mRNA sequences using *OlPax2.2* and *Danio rerio Pax2.1* as queries. There are only two ESTs (AM320053 and AM321390) for *OlPax2.1* that contain the first coding exon, as well as the complete exon 2 and part of exon 3, encoding for N- and C- paired subdomains, respectively. Using the genomic scaffolds and available ESTs collectively (Table S1), an almost complete *OlPax2.1* gene was reconstructed (Figure 1). For *OlPax2.2* gene, one cDNA sequence and four ESTs were retrieved (Table S1), comparison of which revealed both 5′ and 3′ alternatively spliced parts of exon 2 encoding for the N-terminal of paired domain (see Figure 1).

Exon-to-exon comparison of *Pax2/5/8* genes between *O. latipes* and *D. rerio*, shows that in principle there is conservation in the sequence, number, size, and borders of exons and some indication for alternatively spliced exons in *OlPax2* genes (light gray boxes in Figure 1), which are not suggested by the available ESTs. Retrieved ESTs encoding both *OlPax2.1* and *OlPax2.2* support a 5′ alternative splicing donor site in exon 2 (Figure 1, Table S1); the insertion is 12 bp long, and results to four additional aminoacids, exactly at the beginning of the paired domain. The same insertion has been reported for *D. rerio Pax2.1* (Lun and Brand, 1998), as well.

**Figure 2:**
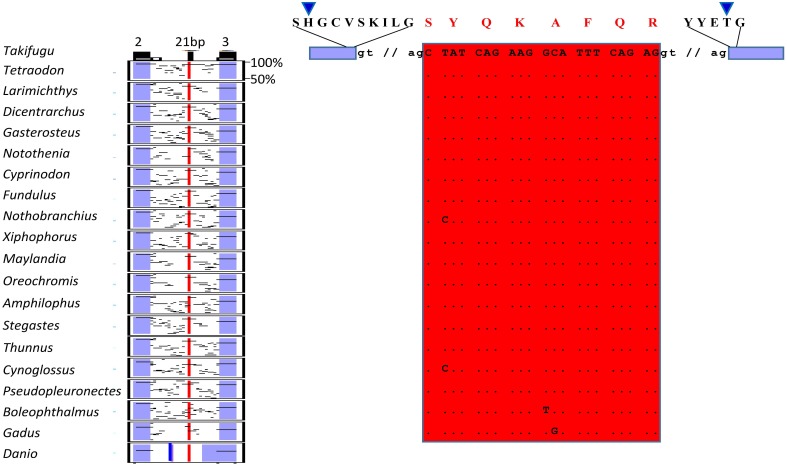
**Alternatively spliced isoform *OlPax2.1(2a+)***. Pip diagram of paired-encoding (2 and 3, blue boxes) and alternatively spliced exon (21 bp, red box) in Acanthomorphata species. Blue thick line represents alternatively spliced exon predicted in three Cyprinidae species. Dots stand for conserved residues, small case letters correspond to intronic nucleotides. Blue arrowheads point to PAI a3 helix.

In the present study we identified two splicing events that lead to insertion of extra aminoacids in the paired domain of *OlPax2* genes. More specifically, an alternatively spliced 21-bp exon was detected between exons 2 and 3 of fish *Pax2.1* genes, which is annotated in some species (e.g., *Poecilia reticulata* and *Maylandia zebra*). This exon could not be detected *in silico* in the genome of *O. latipes*, due to the fact that the intron between exons 2 and 3 is not sequenced. Through BLAST searches and *de novo* analysis of *Pax2.1* genes we spotted this exon in numerous representatives from different orders of Acanthomorphata (Table S2), while PipMaker alignment reveals a high degree of sequence conservation among compared species (Figure 2). A putative exon with proper splice sites has been identified in the respective genomic region of three Cyprinidae species (*D. rerio*, *Pimephales promelas, and Cyprinus* carpio). Even though this exon is highly conserved in these species, the encoded aminoacids are quite dissimilar from those of the Acanthomorphata 21-bp exon (Figure 2). In both cases, inclusion of this exon leads to an alternative transcript, which incorporates seven extra aminoacids toward the end of the a3 helix of the PAI subdomain (Figure 2).

**Figure 3:**
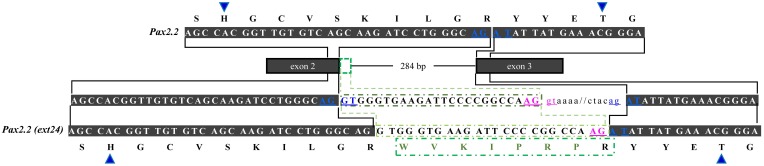
**Alternatively spliced isoform *OlPax2.2(ext24+)***. Dark gray boxes represent constitutive exons, green-outlined box shows part of intron 2 (24 bp) retained in *OlPax2.2*. Thin line represents intron 2 and intronic sequences are in small case letters. Canonical splice sites are in blue letters. Blue arrowheads point to PAI a3 helix. Green letters represent the extra aminoacids incorporated in *OlPax2.1(ext24+)*, when the downstream alternative splicing donor (pink letters) is used.

In regard to *OlPax2.2*, the available mRNA sequence in GenBank and our analysis revealed a 24-bp in-frame extension at the 3′ end of exon 2 (Figures 1, 3, Table S1), which does not alter the downstream translation (Figure 3). This isoform, to which we will refer as *OlPax2.2(ext24+)*, is due to an alternative splicing donor downstream the canonical one (Figure 3). In *D. rerio*, a similar isoform is neither supported by splicing prediction software nor by available mRNA sequences.

It should be noted that splicing prediction analysis of *OlPax2.1* gene revealed the presence of an alternative donor site in exon 2, upstream the canonical one. Deletion of 35 bp at the 3′ end of exon 2 causes a frameshift and leads consequently to a premature stop codon at the beginning of exon 3 (Figure S1). A similar donor site was not *in silico* identified in *D. rerio* (data not shown).

### Comparative analysis of 5a-exon insertion in Pax6 paired domain

In teleosts and tetrapods studied so far, the major part of Pax6 paired domain is encoded by two exons, responsible for the N- and C-subdomains, with a size of 131 and 216 bp, respectively. In Tetrapoda and in the *Pax6.1* copy of teleosts, a small exon of varying size (36–42 bp), namely 5a, has been shown to be included in alternative transcripts, causing an in-frame insertion in the paired domain (Ravi et al., 2013). We wanted to identify at which point of evolutionary history this exon appeared and investigate a putative correlation of the appearance of this exon with the exon/intron organization of *Pax6* homologs. For this reason, already annotated *Pax6* homologs were collected from public databases and available genomes and EST sequences from non-jawed vertebrates, cephalochordates, tunicates, hemichordates, echinoderms, as well as *Drosophila* and *C. elegans* were analyzed (Table S3).

Analysis of a genomic scaffold from *Leucoraja erinacea*, containing the *Pax6* ortholog, provides evidence that besides Holocephali (Ravi et al., 2013), the 5a exon is also present in Elasmonbranchii, the second subclass of Chondricthyes. Unfortunately, no genomes from hagfishes are publicly available, yet the two *Pax6* mRNA sequences that were retrieved from *Eptatretus bergeri* (Table S3), do not provide any indication of an exon5a-like insertion in the paired domain.

In regard to Hyperoartia, genomic scaffolds containing parts of the *Pax6* genes from *Petromyzon marinus* and *Lethenteron japonicum* were retrieved and analyzed, along with two *Pax6* mRNA from the species *L. japonicum* and *Lampetra fluviatilis*. Apparently, there are more than one *Pax6* genes in the *L. japonicum* genome, yet the low genome coverage for both *P. marinus* and *L. japonicum* (5× and 20×, respectively) does not allow for safer conclusions. In all cases, there is no evidence for the existence of the exon 5a in Hyperoartia.

Existing models for *Stongylocentrotus purpuratus* predict two truncated neighboring Pax6 proteins that contain either the paired or the homeobox domain. Taking into account the provided information in SpBase mentioning that the gene models are incomplete and an intervening sequence appears to be missing in a scaffold gap in the Spur3.1 assembly (Howard-Ashby et al., 2006; Cameron et al., 2009), we re-evaluated the prediction and tried to re-construct the *SpPax6* homolog.

**Figure 4:**
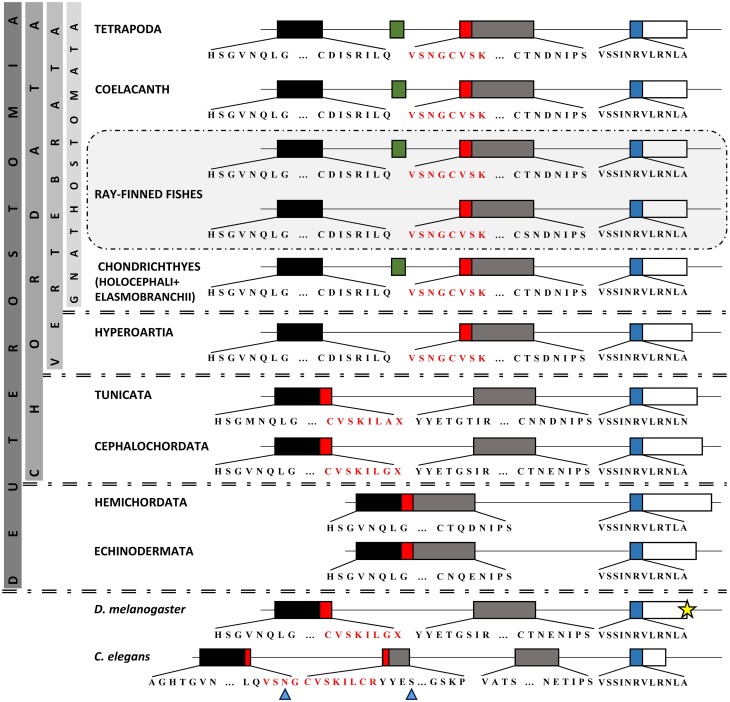
**Comparison of Pax6 paired domain among representatives from Protostomia and Deuterostomia**. Genomic organization of paired domain-encoding exons. Boxes represent exons, thin lines represent introns (the latter are not drawn to scale). Different colors of exons correspond to different subdomains. Green box represents the previously reported exon 5a (Walther and Gruss, 1991). Red box represents the section of paired domain that underwent re-arrangement throughout evolution. For practical reasons, the third exon (665 bp) in *Drosophila* is not depicted to its full length (yellow star). The residue X reveals an asymmetrical exon. Blue arrowheads point to PAI a3 helix.

Our focus was on the exon-intron structure in the region of paired domain (Figure 4). It is apparent, from our analysis, that the size and borders of the two exons encoding the main part of paired domain underwent various changes in different taxonomic groups (Figure 4). More specifically, in basal deuterostomes, such as Echinodermata and Hemichordata, there is one large exon with a size of 347 bp encoding for the first 115 aminoacids of the paired domain. Therefore, there is no intervening non-coding region in the respective position to vertebrate's intron, or in other words “space” for insertion of an alternatively spliced exon in the paired domain. In cephalochordates and tunicates, this large exon has split into two exons, first of which has a size of 166 bp, still larger than the N-subdomain encoding exon in vertebrates, and the second one is 181 bp long, slightly smaller than the respective exon in vertebrates. The 166 bp exon encodes a peptide including the first four aminoacids of a3 helix of the N-subdomain (Xu et al., 1995) and ends shortly after the position where the exon 5a is inserted, i.e., downstream of a3 helix. Thorough *in silico* search in the intronic sequence flanked by the paired-encoding exons in tunicates and cephalochordates did not reveal a putative alternatively spliced exon cassette similar to the exon 5a (Figure 4, Figure S2). It seems that paired-encoding exons obtained fixed borders and size of 131 and 216 bp for PAI and RED, respectively before diversification of cyclostomes and preserved them throughout vertebrates, nonetheless “birth” of exon 5a probably appeared in Gnathostomata (Figure 4, Figure S2). It should be noted that the major re-arrangements concern the genomic region encoding for the PAI a3 helix, in contrast to the high conservation observed toward the C-subdomain.

### Developmental expression of PAI-RED isoforms

**Figure 5:**
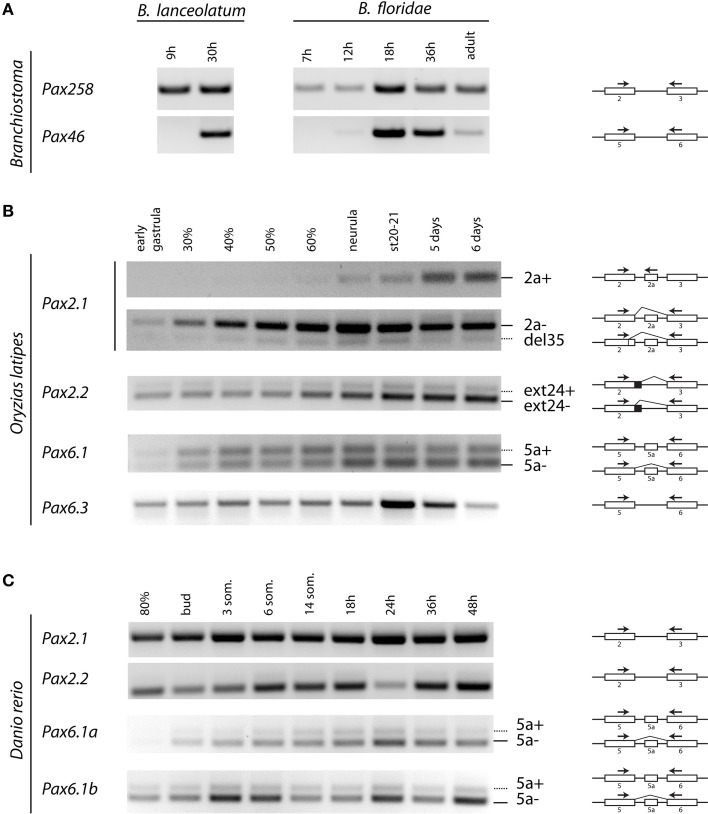
**Temporal expression of *Pax2* and *Pax6* genes during development of *Branchiostoma lanceolatum, B. floridae, Oryzias latipes*, and *Danio rerio* embryogenesis**. cDNA sequences were amplified across the paired encoding exons, as indicated by arrows. **(A)** For *B. lanceolatum* and *B. floridae*, single isoforms were detected for *Pax258* and *Pax46* genes. **(B)** For *O. latipes Pax2.1*, an isoform containing exon 2a was detectable from neurula stage. *Pax2.1(del35)* and *Pax2.2(ext24+)* as well as Pax6.1(5a) isoforms are expressed across all developmental stages. Pax6.3 gene does not possess exon 5a. **(C)** No alternatively spliced isoforms were detected in the case of *D. rerio Pax2.1* and *Pax2.2* genes. Isoforms including exon 5a are present for both *Pax6.1a* and *Pax6.1b* genes.

To verify *in silico* predicted alternative splice isoforms (Figures 2–4, Figure S1) and compare their expression across various developmental stages, we performed RT-PCR using RNA from different embryonic stages of *Branchiostoma lanceolatum, B. floridae, Oryzias latipes*, and *Danio rerio* (Figure 5).

In agreement with our *in silico* analysis, RT-PCR (Figure 5) and DNA sequencing (data not shown) using primers located in exons encoding PAI and RED domains revealed that *B. lanceolatum* and *B. floridae* express single *Pax258* and *Pax46* isoforms.

Three splice isoforms of *OlPax2* genes have been *in silico* predicted. Lack of information concerning intron 2 of *OlPax2.1* prohibited the *in silico* detection of the 21-bp exon, characteristic of Acanthomorphata. However, the presence of 21-bp exon in the *O. latipes* genome was experimentally verified, using a proper set of primers (Table 1), one of which was specifically designed on the 21-bp exon characteristic of Acanthomorphata (Figure 2). Expression of the alternatively spliced *OlPax2.1(2a+)* isoform was detectable from neurula and later developmental stages (Figure 5). In contrast, the truncated isoform *OlPax2.1(del35)*, which results from a deletion in exon 2 and a premature stop codon in the 3rd exon (Figure S1), was present across all developmental stages (Figure 5). *OlPax2.1(del35)* was expressed at much lower level than dominant *OlPax2.1(2a-)* isoform. Sequencing of this isoform revealed that it makes use of the alternative donor site in exon 2, as predicted by *in silico* analysis (Figure S1). The extended isoform *OlPax2.2(ext24+)* (Figure 3) was present at detectable level throughout the examined stages (Figure 5). In the case of *D. rerio Pax2.1* and *Pax2.2* genes, no alternative splice variants were detected, in agreement with splicing prediction analysis.

The alternatively spliced isoform *OlPax6.1(5a-)* was expressed approximately at the same level as isoform *OlPax6.1(5a+)* (Figure 5). In agreement with previous studies (Ravi et al., 2013), *O. latipes* Pax6.3 gene does not possess the equivalent of exon 5a. Variants bearing exon 5a were observed for both *D. rerio Pax6.1a* and *Pax6.1b* genes, and in both cases, expression level of isoform *5a-* was relatively higher than isoform *5a+*.

*In silico* and experimental data, collectively, demonstrate increased complexity of splicing events in vertebrate paired domain of *Pax* genes in comparison to cephalochordates.

## Discussion

Paired box (*Pax*) genes encode for transcription factors that are considered key players in organogenesis and embryonic development. The presence of *Pax* genes in a variety of organisms and the evolution of the PAX family has been the object of various studies (Hill et al., 2010; Paixao-Cortes et al., 2013; Ravi et al., 2013). Whole-genome duplications as well as lineage-specific gene duplications provide additional possibilities for diversified evolution and/or speciation (Bergthorsson et al., 2007; Maere and Van De Peer, 2010). These processes are considered to have played important role in shaping the number of *Pax* homologs in various taxonomic groups (see Paixao-Cortes et al., 2013; Ravi et al., 2013 and references therein), but same applies for alternative splicing, a posttranslational mechanism that also promoted evolution and complexity of Pax proteins (Glardon et al., 1998; Short et al., 2012).

In the present study, we wanted to evaluate the degree of alternative splicing taking place in various lineages, as well as to identify splicing events that are either evolutionary conserved or characteristic of cephalochordates and not vertebrates or vice versa. For this purpose, we collected annotated homologs from Pax2/5/8 and Pax4/6 classes from public databases (NCBI, Ensembl, UCSC, JGI and SpBase). Furthermore, we analyzed *de novo* genomes, ESTs and mRNA sequences from different species, in order to enrich our dataset with taxonomic groups not present in previous studies. Our second focus was *Pax* isoforms from cephalochordates and vertebrates, that differ in the paired domain, and their expression patterns across different developmental stages.

Apart from partially reconstructing the *Oryzias latipes Pax2.1*, using available scaffolds and EST sequences, we identified three new splicing events in the *Pax2* genes of *O. latipes.* The *OlPax2.1(2a+)* isoform is reminiscent of the *5a* isoform found in *Pax6* homologs (Ravi et al., 2013, this study), as it incorporates a 21-bp in-frame-exon located in the intron between the two exons encoding for the paired domain. Our analysis showed that this exon is present in numerous species from various orders of Acanthomorphata and exhibits a high degree of conservation among compared species (Figure 2). We presume that sequence conservation of this mRNA splice form over a wide phylogenetic distance also implies conservation of this isoform's function. A similar exon in terms of location, yet quite divergent in terms of sequence, was *in silico* predicted only in three Cyprinidae species (*D. rerio*, *Pimephales promelas*, and *Cyprinus carpio*).

The second alternatively spliced isoform, namely *OlPax2.2(ext24+)*, results from the use of an alternative splice donor downstream the canonical one at the end of *OlPax2.2* exon 2. In this case, extra aminoacids are incorporated in the middle of a3 helix of the PAI subdomain, with no influence on the downstream sequence. A similar isoform could not be detected in *D. rerio*, neither experimentally nor *in silico*. The sequences surrounding the normal splice junctions of exon 2-intron 2 are highly conserved between *D. rerio* and *O. latipes Pax2.2* genes, yet there is no proper donor-acceptor site in the region of *D. rerio* (AG-AG) that corresponds to the alternative splice site of *O. latipes*.

Both *OlPax2.1(2a+)* and *OlPax2.2(ext24+)* transcripts bear an insertion in the recognition a3 helix of PAI subdomain. Previous studies on insertions in the paired domain of *Pax* genes have proven that, regardless of the number of the inserted aminoacids, disruption of this helix, which is responsible for all major groove DNA contacts of the N-terminal subdomain (Xu et al., 1995, 1999) is expected to inactivate the DNA-binding function of the N-terminal HTH motif, which subsequently leads to severe restriction in the DNA-binding sequence specificity of the paired domain (Kozmik et al., 1997).

The importance of alternative splicing as a mechanism for divergent evolution is established. In the case of *Pax* genes, the fact that insertions in the paired domain may preferentially guide Pax proteins, namely Pax6(5a) and Pax8(S), to the control region of genes containing a modified binding site (5aCON-like sequence, Kozmik et al., 1997), in other words insertions add new target-genes in the repertoire of genes controlled by *Pax* genes, may be indicative of a mechanism through which alternative splicing contributes to the increase of complexity at the level of protein function.

The isoform *OlPax2.1(del35)* makes use of an alternative 5′ splicing donor, upstream of the normal splicing site in exon 2 (N-terminal of paired domain). As mentioned before, the exact junction sequence between exon 2 and intron 2 is not known, nevertheless, the sequence at the normal end of exon 2 (CAG) is in agreement with the optimal consensus for 5′ splice sites (Stephens and Schneider, 1992), in contrast to the sequences at the alternative upstream 5′ splice donor (CGG/GT, Figure S1). As it has been observed before for the *Pax8* gene (Kozmik et al., 1997), there is a higher abundance and constitutive splicing of the *Pax2* mRNA relative to the alternative transcript (Figure 5), a fact that could be attributed to different affinities by which the spliceosomes may recognize the two 5′ donor sites. A similar truncated isoform could not be detected neither during *Pax2.1* transcript analysis of *D. rerio*, nor by *in silico* analysis.

The alternative isoform *OlPax2.1(del35)*, lacks the greater part of a3 helix of PAI domain and ends at a premature stop codon exactly at the beginning of exon 3. Truncated isoforms are not a rare phenomenon, given the fact that approximately 35% of alternatively spliced human transcripts have been found to contain a premature termination codon, rendering them as candidates for non-sense- mediated decay (Green et al., 2003; Lewis et al., 2003). It has been proposed that most low copy number alternative isoforms produced in human cells are likely to be non-functional, therefore we assume that this is also the case for *OlPax2.1(del35)*. Deletion of a3 helix has been observed in one of the Pax6 isoforms in *B. floridae* (Glardon et al., 1998), yet this deletion does not influence downstream translation and hence its functionality. A 32 bp deletion in mouse is responsible for splotch phenotype in mouse (Epstein et al., 1991). In addition, there are accumulating reports about heterozygous deletions of parts of PAI subdomain in general or a3 helix in specific, most of which cause a frame shift and a premature stop codon (Schimmenti et al., 1997; Fletcher et al., 2005) and are correlated with diseases in human (e.g., renal-coloboma syndrome, oligomeganephronia).

In regard to the Pax6 class and the alternative splice isoform *Pax6(5a)*, our analysis showed that an important re-arrangement of coding and non-coding sequences in the region of paired domain took place during evolution. Although conservation of the position of introns has been noted between highly divergent eukaryotes, the number and placement of the majority of introns are dynamically fluctuating during evolution (Hartung et al., 2002; Rogozin et al., 2003). In Hemichordata and Echinodermata, the exon-intron organization does not allow for any type of insertions in the paired domain. In other lineages compared, paired domain is encoded by exons disrupted by one or more introns. Incidents of intron gain and loss as well as intron sliding have been reported for various genes (Hartung et al., 2002), whereas the intron density, i.e., the average number of intron per gene does not necessarily coincide with the position of the genome on the evolutionary tree (Jeffares et al., 2006). We assume that the 5a insertion is characteristic of Gnathostomata. Introns are required for alternative splicing and alternative splicing increases the size of the proteome, thus increasing the level of complexity in higher eukaryotes. Moreover, introns have been found to harbor many conserved non-coding elements, necessary for gene regulation (Irvine et al., 2008; Bhatia et al., 2014).

Unique isoforms were detected during expression pattern study of *Branchiostoma Pax258* and *Pax46* genes. This is in agreement with *in silico* analysis, during which no splicing events involving the paired domain were predicted. In contrast, new alternative spliced variants were identified for fish species. Previous studies have shown that there is no developmental regulation of paired domain alternative splice forms of *Pax6* and *Pax8*, as opposed to splicing events affecting the C-terminal sequences of Pax8 protein (Kozmik et al., 1993, 1997). In principle, non-constitutive *OlPax2* isoforms are expressed at low levels, therefore at this stage, it is not easy to conclude as to the regulation of these isoforms. Nonetheless there is an indication of a temporal regulation of *OlPax2.1(2a+)* isoform, which requires further investigation.

### Conflict of interest statement

The authors declare that the research was conducted in the absence of any commercial or financial relationships that could be construed as a potential conflict of interest.
