# Supplementary material for: Pax2/5/8 and Pax6 alternative splicing events in basal chordates and vertebrates: a focus on paired box domain
Source: Front Genet. 2015 Jul 2;6:228. doi: 10.3389/fgene.2015.00228 (PMC4488758; doi:10.3389/fgene.2015.00228)
Supplement: Supplementary file 1 [file Table1.PDF]

Table S1. Accession numbers of Pax2 related mRNA, ESTs and genomic sequences for species included in the comparative analysis in Figure 1.

Species	Gene	mRNA	ESTs	Genomic records
Mus musculus	**Pax2**	BC150484, BC148232		ENSMUSG00000004231
	**Pax5**	M97013, BC156134, BC157081	AI173210, CA465006, BY222280, BF013073	ENSMUSG00000014030
	**Pax8**			ENSMUSG00000026976
Homo sapiens	**Pax2**	L25597, M89470	DB077633, BJ989901	ENSG00000075891
	**Pax5**	M96944, AY463952-AY463957, FJ626415- FJ626425, AF080573	AW406299, AI762480	ENSG00000196092
	**Pax8**	X69699, S77904-S77906	AU141429, AU141907	ENSG00000125618
Danio rerio	**Pax2.1**	AF067530-AF067541	CA471205	ENSDARG00000028148
	**Pax2.2**	BC162214, AF072547		ENSDARG00000032578
	**Pax5**	AF072548	CO353049, FP141998	ENSDARG00000037383
	**Pax8**	AF072549	FP184042, CD588170, FP119188, FP106520	ENSDARG00000015879
Oryzias latipes	**Pax2.1**		AM320053, AM321390	NW_004088010, NW_004093539
	**Pax2.2**	Z97020	AM307102, DK010940, AM356055, DK033442	NC_019877
	**Pax5**		BJ017365, AM141546	NC_019859
	**Pax8**		AM297532, AM320946, AJ457242	NW_004087989, NW_004095010
Branchiostoma floridae	**Pax258**	AF053762, AF053763	BW740265	NW_003101569.1
