# Supplementary material for: Pax2/5/8 and Pax6 alternative splicing events in basal chordates and vertebrates: a focus on paired box domain
Source: Front Genet. 2015 Jul 2;6:228. doi: 10.3389/fgene.2015.00228 (PMC4488758; doi:10.3389/fgene.2015.00228)
Supplement: Supplementary file 2 [file Table2.PDF]

Table S2. Acanthomorphata species bearing the 21-bp exon in their *Pax2.1* gene (see Figure 2) and accession numbers of genomic sequences containing these genes

Species	Genomic sequence AC	Species	Genomic sequence AC
Amphilophus citrinellus	CCOE01000564.1	*Pampus argenteus*	JHEK01068794.1
Anoplopoma fimbria	AWGY01157159.1	*Periophthalmus magnuspinnatus*	JACL01008226.1
Boleophthalmus pectinirostris	JACK01029173.1	*Periophthalmodon schlosseri*	JACM01076439.1
Cynoglossus semilaevis	NW_007584308.1	*Poecilia formosa*	NW_006799951.1
Cyprinodon nevadensis pectoralis	JSUU01000555.1	*Poecilia reticulata*	NW_007614917.1
Cyprinodon variegatus	JPKM01000629.1	*Pseudopleuronectes yokohamae*	BBOV01038679.1
Dicentrarchus labrax	CBXY010000978.1	*Pundamilia nyererei*	NW_005187550.1
Fundulus heteroclitus	JXMV01114947.1	*Scartelaos histophorus*	JACN01150698.1
Gadus morhua	CAEA01473814.1	*Sebastes nigrocinctus*	AUPR01102968.1
Gasterosteus aculeatus	AANH01005104.1	*Sebastes rubrivinctus*	AUPQ01101565.1
Haplochromis burtoni	NW_005179406.1	*Stegastes partitus*	NW_007577864.1
Larimichthys crocea	NW_011322448.1	*Takifugu flavidus*	AOOT01011690.1
Maylandia zebra	NW_004531737.1	*Takifugu rubripes*	NC_018893.1
Neolamprologus brichardi	NW_006272006.1	*Tetraodon nigroviridis*	CAAE01014563.1
Nothobranchius furzeri	JNBZ01239218.1	*Thunnus orientalis*	BADN01007783.1
Notothenia coriiceps	NW_011340598.1	*Xiphophorus maculatus*	NW_005372243.1
Oreochromis niloticus	NT_167486.1		
