# Supplementary material for: Pax2/5/8 and Pax6 alternative splicing events in basal chordates and vertebrates: a focus on paired box domain
Source: Front Genet. 2015 Jul 2;6:228. doi: 10.3389/fgene.2015.00228 (PMC4488758; doi:10.3389/fgene.2015.00228)
Supplement: Supplementary file 3 [file Table3.PDF]

Table S3. Accession numbers of Pax6 related mRNA, ESTs and genomic sequences for species included in the comparative analysis in Figure 4. NA: no Pax6-related sequences are available in public databases

Species	mRNA	ESTs (total available ESTs)	Genomic records
**Tetrapoda**			
Homo sapiens	AB593092- AB593094, M77844	BY795116.2, DA571138.1	ENSG00000007372
**Coelocanth**			
Latimeria chalumnae	NA	NA (0)	NW_005819101
**Ray-finned fishes**			
Danio rerio Pax6.1a	X63183, X61389, BC076068	CN318631, FP158091	NC_007136.6
Danio rerio Pax6.1b	BC076068, AF061252	CT604580, CK396395	NC_007118.6
Oryzias latipes Pax6.1	AJ000938	DK094078, AM316636	NW_004087942
Oryzias latipes Pax6.3	NA	BJ013007, AM298948	NC_019864.1
**Chondrichthyes**			
Callorhinchus milii	NA	NA (109,965)	NW_006890061.1
*Leucoraja erinacea**	NA	NA (31,167)	AESE010059308, AESE010129024.1
**Hyperoartia**			
Petromyzon marinus	NA	NA (120,731)	AEFG01058654.1
Lethenteron camtschaticum	AB061220	NA (8,788)	APJL01012232, APJL01012235, APJL01012244, APJL01012245, APJL01012247, APJL01012248, APJL01047766, APJL01047767, APJL01047768, APJL01115859, APJL01166332, APJL01166741
Lampetra fluviatilis	AF384973 (partial)	NA (6)	NA
**Hyperotreti**			
Eptatretus bergeri	FR720087, AB270704	NA (27,909)	NA
**Tunicata**			
Ciona intestinalis	NM_001032469	BW222431, BW415232 BW087598, FF702282 BW360858	NW_004190331.2

Species	mRNA	ESTs (total available ESTs)	Genomic records
Ciona savignyi	NA	NA (84,302)	AACT01018212.1
**Cephalochordata**			
Branchiostoma floridae#	AJ223440, AJ223441, AJ223442, AJ223443, AJ223444	NA (334,502)	NW_003101385
Branchiostoma belcheri	NA	NA (24,836)	DQ991503
Branchiostoma lanceolatum	NA	NA (0)	lcl|xpSc0039676
**Hemichordata**			
Saccoglossus kowalevskii	AY313154	FF491918, F449641, FF479505, FF479254	NW_003122423
**Echinodermata**			
*Lytechinus variegatus**	NA	NA (1)	AGCV02304654
Metacrinus rotundus	GU812278	NA (0)	NA
Paracentrotus lividus	U14621	NA (140,897)	NA
*Patiria miniata**	HQ128717	NA (1,249)	AKZP01060555
Strongylocentrotus droebachiensis	DQ230536	NA (0)	NA
Strongylocentrotus intermedius	KF733999	NA (66)	NA
Strongylocentrotus purpuratus#	NA	NA (141,833)	NW_011994489.1, AAGJ05116494
**Protostomia**			
Drosophila melanogaster	X79493, BT025949	EL874785-EL874786, EL874913, EL875029	NC_004353.4
Caenorhabditis elegans	U31537, U29145, U29184	CB391511, AU205038, CB391537, AU208824, CB391547, BJ113838	NC_003284.9

Genome annotation concerning *Pax6* genes was re-evaluated

* Species for which genomic scaffolds were analyzed and annotated *de novo*
