# Supplementary material for: Pax2/5/8 and Pax6 alternative splicing events in basal chordates and vertebrates: a focus on paired box domain
Source: Front Genet. 2015 Jul 2;6:228. doi: 10.3389/fgene.2015.00228 (PMC4488758; doi:10.3389/fgene.2015.00228)
Supplement: Supplementary file 5 [file Image2.PDF]

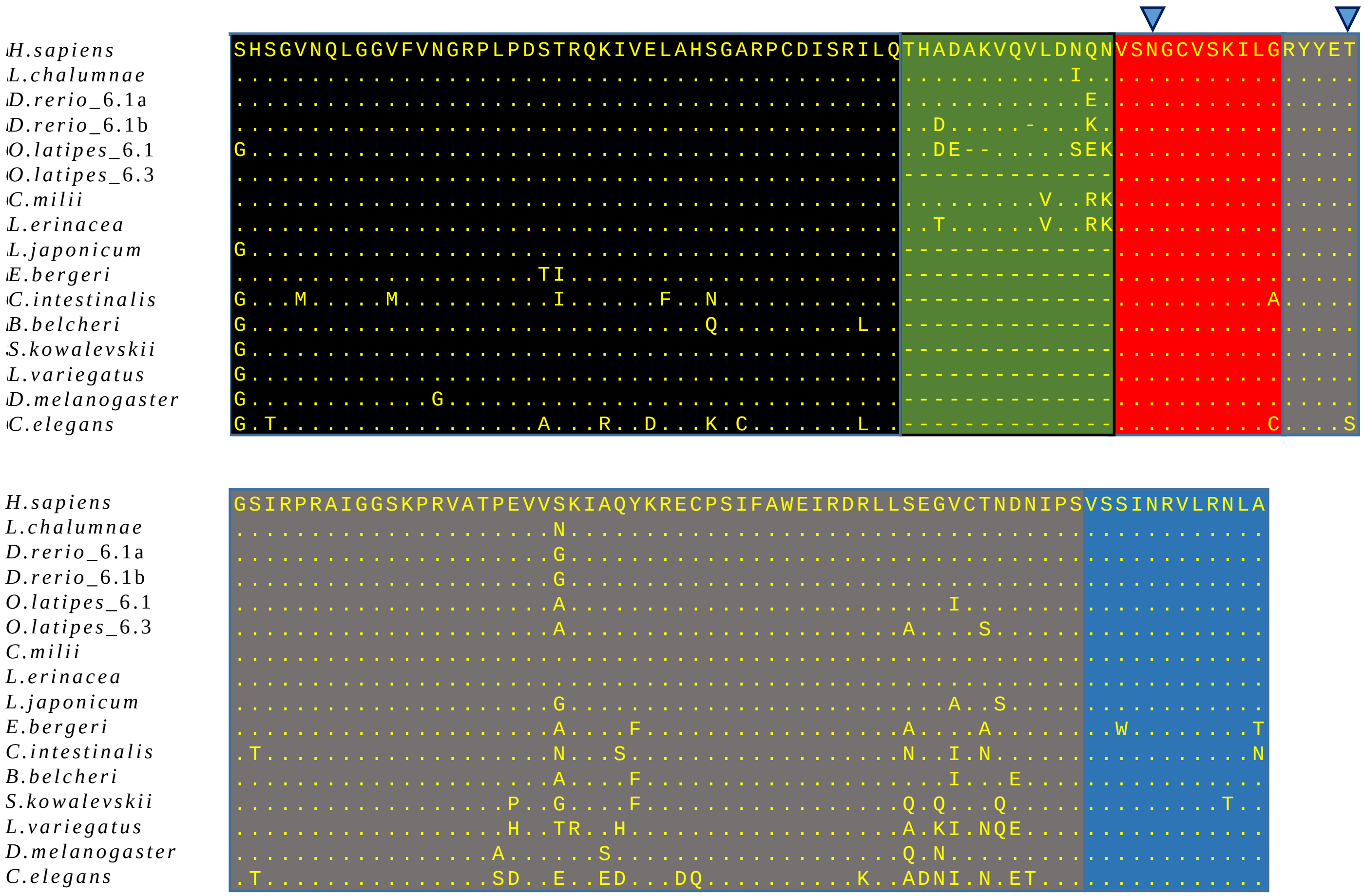

Figure S2. Alignment of Pax6 paired domain among representatives from Protostomia and Deuterostomia phyla. Shading is according to exons in Figure 4. Dots and dashes stand for conserved or missing residues, respectively. Blue arrowheads point to PAI a3 helix.
