# Supplementary material for: Pax2/5/8 and Pax6 alternative splicing events in basal chordates and vertebrates: a focus on paired box domain
Source: Front Genet. 2015 Jul 2;6:228. doi: 10.3389/fgene.2015.00228 (PMC4488758; doi:10.3389/fgene.2015.00228)
Supplement: Supplementary file 4 [file Image1.PDF]

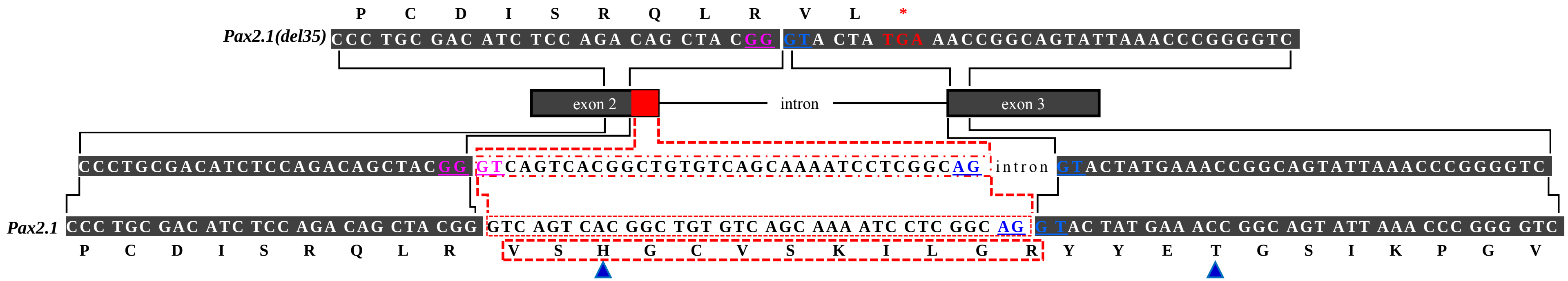

Figure S1. Alternatively spliced isoform *OlPax2.1(del35)*. Dark grey boxes represent constitutive exons, red box shows part of exon 2 (35 bp) deleted in *OlPax2.1*. Thin line represents intron 2. Intron sequence is not known, due to a non-sequenced region in *O. latipes* genome. Canonical splice sites are in blue letters. Red star represents the premature stop codon caused due to the use of an alternative 5´ splice donor (pink) and the subsequent frameshift. Blue arrowheads point to PAI a3 helix.
